# Supplementary material for: Magnetic Structure and Origin of Insulating Behavior in the Ba2CuOsO6 System, and the Role of A-Site Ionic Size in Its Bandgap Opening: Density Functional Theory Approaches
Source: Nanomaterials (Basel). 2021 Dec 31;12(1):144. doi: 10.3390/nano12010144 (PMC8746825; doi:10.3390/nano12010144)
Supplement: Supplementary file 1 [file nanomaterials-12-00144-s001.zip › nanomaterials-1514855-supplementary.pdf]

Supporting Information

Magnetic Structure and Origin of Insulating Behavior in the Ba_2CuOsO_6 System, and the Role of A-Site Ionic Size in Its Bandgap Opening: Density Functional Theory Approaches

Taesu Park[1,†], Wang Ro Lee[2,†], Won-Joon Son[3], Ji-Hoon Shim[1,4,*], Changhoon Lee[5,*]

[1] Department of Chemistry, Pohang University of Science and Technology, Pohang, 37673, Korea

[2] Faculty of Liberal Education, Jeonbuk National University, Jeonju 54896, Korea

[3] Samsung Advanced Institute of Technology (SAIT), Samsung Electronics, 130 Samsung-ro, Yeongtong-gu, Suwon 16678, Korea

[4] Division of Advanced Materials Science, Pohang University of Science and Technology, Pohang, 37673, Korea

[5] Max Planck POSTECH Center for Complex Phase of Materials, Pohang University of Science and Technology, Pohang, 37673, Korea

Table S1. The expected electronic properties for various U sets obtained from DFT+U+SOC calculation.

	Metallic	Insulating
U_{Os}=2, U_{Cu}=3 eV	O	
U_{Os}=3, U_{Cu}=4 eV	O	
U_{Os}=4, U_{Cu}=5 eV	O	
U_{Os}=3, U_{Cu}=2 eV	O	
U_{Os}=4, U_{Cu}=3 eV	O	
U_{Os}=5, U_{Cu}=4 eV		O

Table S2. Values of the coefficients n_1 – n_8 of Equation (1) for the nine ordered spin states (FM and AF1 – AF8) to extract spin exchange interactions J_1 – J_8.

	n_1	n_2	n_3	n_4	n_5	n_6	n_7	n_8
FM	–4	–2	–2	–2	–4	–4	–2	–2
AF1	4	2	–2	–2	–4	–4	–2	–2
AF2	–4	2	–2	–2	4	4	–2	–2
AF3	0	0	–2	2	–4	0	–2	–2
AF4	0	0	2	–2	0	–4	–2	–2
AF5	0	0	–2	–2	–4	4	–2	–2
AF6	0	0	–2	–2	4	–4	–2	–2
AF7	0	0	–2	0	–4	0	–2	2
AF8	0	0	0	–2	0	–4	2	–2

Table S3. The optimized structural parameters of A_2CuOsO_6 (A=Ba, Sr, and Ca) obtained from the DFT+U+SOC (U_{Cu} = 4, U_{Os} = 5 eV). Space group I4/m (#87). A is at site (0, 0.5, 0.25), Cu at (0, 0, 0), Os at (0, 0, 0.5), O_{ab} at (x, y, 0) and O_c at (0, 0, z).

	**Ba_2CuOsO_6**	**Sr_2CuOsO_6**	**Ca_2CuOsO_6**
a (Å)	5.6104	5.4037	5.2267
c (Å)	8.4920	8.4070	8.3552
a/c ratio	0.66	0.64	0.63
O_{ab} x	0.2568	0.2113	0.3284
O_{ab} y	0.2491	0.2941	0.1766
O_c z	0.7255	0.7313	0.7358
Os-O_{ab} (Å)	1.961	1.916	1.914
Os-O_c (Å)	1.915	1.945	1.970

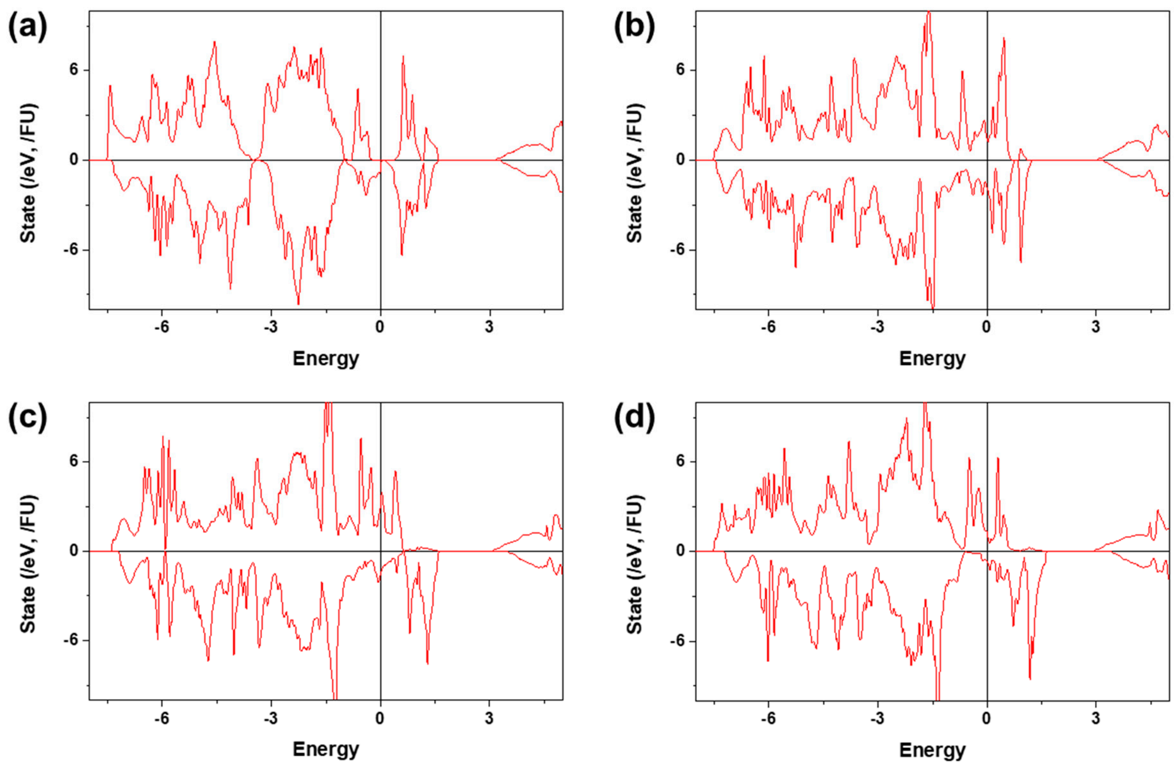

Figure S1. Total density of states in Ba_2CuOsO_6 calculated by DFT+U+SOC. U values for Cu and Os in this DOS calculation are chosen as (a) U_{Cu} = 4 eV, U_{Os} = 5 eV, (b) U_{Cu} = 5 eV, U_{Os} = 4 eV, (c) U_{Cu} = 3 eV, U_{Os} = 4 eV, and (d) U_{Cu} = 2 eV, U_{Os} = 3 eV.
